# Diseases of the Arteries, Veins, and Capillaries

**Published:** 1892-01-16

**Authors:** 


### THE PRACTITIONER'S MIRROR AND RETROSPECT.

[*The Editor will be glad to receive offers of co-operation and contributions from members of the profession. All letters should be addressed to* THE EDITOR, THE LODGE, PORCHESTER SQUARE, LONDON, W.]

### MEDICINE.

### DISEASES OF THE ARTERIES, VEINS AND CAPILLARIES.

#### II.—THE VEINS.

The morbid conditions of these vessels are generally summed up by saying that they are "very similar to those of arteries." This is only partially true. For example, the various diseases of the capillaries which put a stress upon the arterial system and cause hypertrophy of the muscular coat, do not affect the veins. Atheroma, and other conditions involving the subendothelial tissue are rare. Moreover, the thin, flabby walls are more easily injured; and septic material absorbed from the tissues would, of course, naturally attack the veins first.

The most common and interesting morbid condition distinctive of these vessels is Varix. This appears to begin as an 'endovenitis' or chronic inflammatory thickening of the subendothelial coat. In the early stages this is the only change found, but it is quickly followed by considerable hypertrophy of the muscular wall, and by increase in the adventitia and fibrous tissues surrounding the vessels. The endothelium itself remains until the whole vein is thickened. There is a remarkable increase of small elongated connective tissue cells in the subendothelial coat, which looks under the microscope very like unstriped muscle, and this layer is, after the disease has lasted some time, the thickest of the tunics. The causes of varicose veins are various, long continued standing or walking, tight garters, relaxed or feeble muscles, diminished 'vis a tergo' from weak cardiac action, enlarged glands in the groin, &c., appear to contribute in different individuals. It is not always easy, however, to understand how the result is brought about. The statement, for instance, that the length of limb increases the liability to varicose veins by preventing the return of blood, and counteracting the force of the heart by gravity, is only partially correct, the hydrodynamics of the U-shaped tube, and the law that fluid tends to finds its own level must be taken into account. Again, the condition is almost as common in people who do not use garters as in these who do. Probably the surroundings of the vessel have a great deal to do with the production of varix; fat persons are not so liable to it as thin, and it seems to occur most readily in those whose veins are not firmly supported by strong fascia and fat, &c. Conditions of the fascia in the groin and of the pelvic viscera (accumulations of fæces, &c.) may help to cause the disease.

#### III.—THE CAPILLARIES.

It should be noted that these minute vessels consist of soft endothelial cells, with sufficient "cement" substance to keep them together, and occasionally a perceptible amount of fine connective tissue. This latter sometimes forms a distinct layer, called the *adventitia capillaris* of His. Both the endothelium and fibrous tissue are in organic connection with surrounding parts. In the embryonic condition these capillaries have the power of contractility. The cells of which they are composed easily proliferate, and are very pliable. This last property is seen in the disease called *ectasis*, in which varicose dilitations occur on the capillaries. This condition only occurs apparently as the result of venous obstruction and over-distension.

*Anyloid* disease commences in the "adventitia capillaris" above mentioned, but it extends usually to the fibrous tissue in the arterioles, and to other parts. It occurs as a result of deprivation of certain salts, which are subtracted from the blood by purulent discharges, &c. Kekulé has shown that the substance "lardacein" contains nothing similar to starch; it is possibly an intermediate stage between albumen on the one hand and cholesterin on the other. All the normal proteids of the body exist in a saline medium, and they are modified by the withdrawal of these salts; in fact, it is impos ible to obtain albumen without some admixture of the chlorides and phosphates of the alkalies. But until more is definitely known of the composition of the proteids and their derivatives the nature of Lardacein cannot be ascertained.

It has been stated before that Bright's disease affects the capillaries first and the arteries afterwards. In what way this takes place is doubtful, but probably the vitality of the endothelium is lowered and the blood current retarded, for it is well known, that blood circulates with difficulty through a dead capillary. No microscopic change has been detected in these vessels in kidney disease. In connection with this subject it is interesting to note that Dr. Waymouth Reid has promulgated the theory that the cells forming capillary walls do not merely act as passive agents in the diffusion of *liquor sanguinis*, but have a distinct secretory function. This hypothesis is somewhat strengthened by the discovery that in certain membranes in the cells (such as the frog's skin) "osmosis" goes on much more readily in one direction than another. This, and other ascertained facts point to the conclusion that "filtration" may often be a distinct selective and secretory process. The loss of this property might perhaps explain the production of œdema in the kidney and some forms of heart disease, the physiology of which is not clear. It might also help in elucidating the phenomena of the slowing of the current and ultimate stasis in inflammation.

Inasmuch as the well-being of the tissues depends on the proper transudation of nutritive plasma through the capillary walls, the fact of disease originating in these minute channels is important. Two morbid processes, viz, lardaceous degeneration, and the changes of Bright's disease and arterio-capillary fibrosis appear to commence in them. It is probable, also, that tubercular endarteritis sometimes has its starting point in the blocking of capillaries by bacilli, &c.; this extending to the arterioles.

We may, by way of summary, point out that (1) the fibrous tissue of the subendothelial layer in arteries is the place of origin of most of the diseases of these vessels, and that this is due partly to the nature of this coat, and partly to its proximity to the blood; alterations in this fluid being really the source of most of these affections. (2) That although morbid conditions are rarely found in the capillaries, there is, nevertheless, proof that at least two lesions originate in them; and (3) that the veins owe their immunity from certain diseases to their position in front of the capillaries; and their liability to others (septic conditions, &c.) to the same cause.

